# PCSK9 in Cancer: Biological Mechanisms and Implications for Therapeutic Resistance

**DOI:** 10.3390/biom16010067

**Published:** 2025-12-31

**Authors:** Nuriza Ulul Azmi, Rezi Riadhi Syahdi, Meidi Utami Puteri, Arry Yanuar, Mitsuyasu Kato, Fadlina Chany Saputri

**Affiliations:** 1 Department of Experimental Pathology, Faculty of Medicine, University of Tsukuba, Tsukuba 305-8575, Ibaraki, Japan; 2 Department of Pharmacology and Toxicology, Faculty of Pharmacy, Universitas Indonesia, Kampus UI Depok, Depok 16424, Indonesia; 3 Biomedical Computation and Drug Design Laboratory, Faculty of Pharmacy, Universitas Indonesia, Kampus UI Depok, Depok 16424, Indonesia; 4 National Metabolomics Collaborative Research Centre, Faculty of Pharmacy, Universitas Indonesia, Kampus UI Depok, Depok 16424, Indonesia; 5 Faculty of Pharmacy, Universitas Indonesia, Kampus UI Depok, Depok 16424, Indonesia

**Keywords:** cancer, chemoresistance, hypercholesterolemia, lipid metabolism, PCSK9

## Abstract

Cancer remains a major global health challenge, largely due to its biological heterogeneity and the capacity of tumor cells to adapt under metabolic and environmental stress. Lipid metabolism has increasingly been recognized as a contributor to tumor progression and treatment response. Proprotein convertase subtilisin/kexin type 9 (PCSK9), widely known for regulating low-density lipoprotein (LDL) receptor turnover and systemic cholesterol levels, has recently been implicated in cancer biology. Emerging evidence shows that PCSK9 influences processes such as cell survival, MHC-I-mediated immune recognition, membrane receptor trafficking, and cellular stress responses, indicating roles that extend beyond its canonical metabolic function. These mechanisms also raise the potential relevance of PCSK9 to affect treatment tolerance and drug responsiveness. This review summarizes current knowledge on the biological functions of PCSK9 in cancer and examines how these pathways may have implications for therapeutic resistance.

## 1. Introduction

Cancer remains one of the leading causes of mortality worldwide, and its global incidence is expected to rise over the coming decades [1]. A major challenge in cancer treatment is the development of resistance to anticancer therapies, which accounts for more than 90% of cancer-related deaths and contributes to disease progression, metastasis, and relapse [2]. Resistance of cancer cells to anticancer drugs may arise from intrinsic tumor properties or from acquired resistance during treatment. The following multiple mechanisms have been studied to demonstrate the development of anticancer drug resistance in cancer cells, including increased drug efflux, drug target alteration and mutation, evasion of apoptosis, enhanced DNA repair, epigenetic modification, induction of epithelial–mesenchymal transition (EMT), as well as cancer stemness-associated pathways [3,4,5].

To date, lipid metabolism has emerged as an important factor contributing to tumor progression and therapeutic response. Several studies have demonstrated that altered cholesterol and lipid regulatory pathways can support cancer cell survival and promote resistance to therapy, leading to increasing interest in lipid-modulating strategies as potential anticancer approaches [6]. Notably, combining clinically approved proprotein convertase subtilisin/kexin type 9 (PCSK9) inhibitors with immune checkpoint blockade has shown synergistic antitumor effects in preclinical models, further highlighting the relevance of lipid regulatory pathways in cancer biology [7].

PCSK9 was originally identified and linked to human disease in 2003 through independent functional and genetic studies [8,9]. Early work described the protein as neural apoptosis-regulated convertase-1 (NARC-1), implicating it in cellular differentiation and liver biology, before subsequent genetic analysis established PCSK9 as a causal gene in autosomal dominant familial hypercholesterolemia [8,9]. These genetic studies demonstrated that gain-of-function mutations in PCSK9 lead to marked elevations in circulating low-density lipoprotein cholesterol (LDL-C) [8]. Mechanistic studies subsequently demonstrated that PCSK9 is a key post-translational regulator of lipid metabolism by promoting endosomal/lysosomal degradation of the low-density lipoprotein receptor (LDLR), thereby limiting LDL-C clearance [10,11,12]. These discoveries led to rapid therapeutic development and the clinical approval of PCSK9-targeting agents, including monoclonal antibodies (e.g., evolocumab and alirocumab) and an siRNA-based therapy (inclisiran), for LDL-C lowering [13,14,15]. Cardiovascular outcomes trials further demonstrated cardiovascular risk reduction with PCSK9 monoclonal antibodies in high-risk patients [16,17]. Large clinical trials demonstrated substantial and sustained LDL-C reductions with an overall acceptable safety profile, supporting PCSK9 as a viable therapeutic target in clinical settings and gaining interest in PCSK9 biology in addition to cardiovascular disease, including in cancer-related contexts [18,19,20].

PCSK9 has emerged as a regulator of cancer biology beyond its canonical role in cholesterol metabolism. Elevated PCSK9 expression has been documented in several malignancies, where it influences pathways linked to proliferation, metastatic capacity, and modulation of antitumor immunity [21,22,23,24]. Recent findings also indicate a possible contribution of PCSK9 to the acquisition of therapeutic resistance, including chemoresistance and targeted-therapy tolerance [22,25]. Given these observations, PCSK9 has gained interest as a molecule at the intersection of metabolism, cancer progression, and treatment response. This review summarizes current knowledge on the biological functions of PCSK9 in cancer and examines how these mechanisms may have implications for therapeutic resistance, highlighting its potential as a prognostic marker and emerging therapeutic target.

## 2. The Biological Function of PCSK9 in Lipid Metabolism

PCSK9 is a liver-derived serine protease, belonging to the ninth member of the subtilisin/kexin family, which shares structural features with bacterial subtilisin and yeast kexin protease [26]. The human mRNA PCSK9 encoded a 692 amino acid protein consisting of three distinct domains, a signal peptide, a prodomain, and a catalytic domain, each contributing to its maturation and biological activity [19]. As a key regulator of lipid homeostasis, PCSK9 affects plasma LDL-C levels by binding to the LDLR on the hepatocyte surface. PCSK9 induces the formation of the receptor-ligand complex to lysosomal degradation, thereby reducing LDLR recycling and increasing circulating LDL-C levels [27,28]. Notably, elevated plasma LDL-C is a well-known major risk factor for atherosclerosis and coronary heart disease, suggesting PCSK9 is an important therapeutic target in cardiovascular disease-related therapy [29]. Human genetic study showed that naturally occurring loss-of-function variants in PCSK9 are associated with lifelong reductions in LDL-C and protection from coronary heart disease, supporting clinical validation of PCSK9 as a therapeutic target [30].

In addition to lowering LDL-C, PCSK9 has also been associated with a reduction in other lipid components (apolipoprotein B, lipoprotein(a), non-high-density-cholesterol) and markers of vascular inflammation, with clinical benefits observed in patients with acute coronary syndrome (ACS) [31]. These findings have led to PCSK9 inhibition being recognized as an effective cholesterol-lowering strategy, supported by the development of FDA-approved therapeutics [32]. The first two approved agents as PCSK9 inhibitors, alirocumab, in July 2015 [33] and evolocumab in August 2015 [34], are monoclonal antibodies that prevent PCSK9-LDLR interaction. Recently, a small interfering RNA (siRNA) therapy that suppresses hepatic PCSK9 synthesis, namely inclisiran, received approval in December 2021, offering a novel modality for long-term PCSK9 reduction [35]. The emerging collective reports highlight the central role of PCSK9 in lipid metabolism and provide a rationale for investigating its broader biological functions, including emerging roles in cancer.

## 3. Lipid Metabolism and Its Relevance to Cancer Biology

The regulation of lipid metabolism is now widely recognized as contributing to cancer progression by sustaining tumor growth and survival [36]. Tumor cells rely on lipids as part of their metabolic reprogramming, not only as structural components of membrane synthesis but also as sources of signaling molecules and as substrates for metabolic plasticity or adaptation. To support rapid proliferation, cancer cells increase de novo fatty acid synthesis and enhance lipid uptake, often through oncogenic pathways involving sterol regulatory element-binding proteins (SREBPs) and related lipogenic regulators [37,38,39,40]. Previous studies demonstrate that SREBP-dependent lipogenesis is required for tumor growth and survival in glioblastoma and colon cancer [39,40]. However, under hypoxic conditions, cells shift toward increased fatty acid uptake and lipid droplet accumulation to maintain proliferation and suppress oxidative stress in glioblastoma, rather than activating de novo lipid synthesis [41,42].

Cholesterol metabolism also plays a major role in tumor development. Elevated membrane cholesterol enriches lipid rafts and stabilizes the clustering of oncogenic receptors, thus amplifying downstream signaling through receptor tyrosine kinases such as EGFR and HER2 [43,44]. Beyond proliferative signaling, cholesterol-rich raft organization contributes to immune evasion. Nanoscale imaging studies reveal that PD-L1 is concentrated within cholesterol-rich membrane microdomains and that disrupting these domains alters its nanoscale organization, implicating its immunosuppressive function [45]. Lipid metabolic alterations further potentiate the metastatic phenotypes. Increased fatty acid uptake through CD36 promotes metastasis initiation by fueling mitochondrial β-oxidation, thereby producing ATP and electron carriers -NADH and FADH_2_- needed to support migration, invasion, and survival in distant niches. Inhibition of CD36 markedly suppresses metastasis in vivo [46]. Several cancers have also been reported to accumulate lipid droplets, which act as dynamic reservoirs of neutral lipids that maintain homeostasis under lipotoxic and oxidative stress [41,42]. In addition, they provide metabolic fuel under hypoxic and nutrient-limited conditions to improve survival during dissemination and colonization.

The metabolic adaptation driven by lipids is strongly associated with the therapeutic response and resistance mechanisms. Changes in membrane lipid composition alter membrane order, fluidity, and nanodomain organization, which affect drug partitioning, uptake, intracellular distribution, and efflux, contributing to the acquisition of multidrug resistance [47]. Lipid biosynthetic enzymes, such as fatty acid synthase (FASN), also promote EMT, invasion, and HER-family related signaling, linking lipogenesis to aggressiveness and drug tolerance [48]. Moreover, rewired lipid metabolism, including increased de novo lipogenesis, fatty acid uptake, lipid droplet biogenesis, and fatty acid oxidation (FAO), supports cancer stem cell (CSC) maintenance and resistance to cytotoxic stress, to sustain self-renewal and survival after chemotherapy [49,50].

Given the extensive contribution of lipid metabolic pathways to tumor progression and chemoresistance, molecules that regulate systemic or cellular lipid homeostasis have become increasingly relevant to cancer research. PCSK9, a key regulator of LDLR turnover and cholesterol homeostasis, is suggested to interconnect the lipid metabolism with key pathways governing oncogenic signaling and the tumor immune microenvironment. Recent work indicates that PCSK9 not only controls LDLR-dependent cholesterol regulation but also modulates the immune microenvironment and influences responses to immune checkpoint blockade [7,51]. Thus, understanding how lipid metabolism shapes oncogenic signaling and therapeutic resistance provides an essential foundation for evaluating the emerging roles of PCSK9 in cancer.

## 4. PCSK9 and Cancer

Accumulating evidence shows that PCSK9 contributes to various cancer progression beyond its canonical role in cholesterol homeostasis. PCSK9 dysregulation has been documented across multiple types of cancers, including breast cancer, head and neck squamous cell carcinoma, hepatocellular carcinoma, gastric cancer, lung cancer, colorectal cancer, and many others, where it influences tumor growth, metastasis, immune evasion, and therapy resistance [7]. Table 1 summarizes the research related to PCSK9 and cancer types and the molecular mechanisms underlying these cancer-associated functions of PCSK9 are synthesized and discussed in detail in Section 5.

In parallel with experimental investigations, several bioinformatics and pan-cancer studies from large-scale datasets, including TCGA and cBioPortal-linked resources, have evaluated PCSK9 expression patterns and clinical associations across tumor types. In a TCGA-based pan-cancer analysis, PCSK9 showed heterogenous expression and tumor-specific prognostic associations, with immune-infiltration correlations that varied by cancer type [22]. The studies explore relationships with markers of CD8^+^ T cells, macrophage polarization, and T-cell exhaustion signatures in specific cohorts [22]. More recently, a TCGA-driven study across 33 tumor types integrated immunogenomic correlations with functional experiments and reported that higher PCSK9 expression was associated with more advanced disease stage and poorer prognosis in selected tumors [52]. Meanwhile PCSK9 inhibition improved immune contexture in vivo, including increased dendritic-cell infiltration and increased MHC-II expression, enhancing the efficacy of a peptide vaccine strategy in their model system [52]. Importantly, TCGA stratification can also connect PCSK9 to defined oncogenic contexts, as analysis of TCGA COAD/READ cohorts in colorectal cancer stratified by APC loss and KRAS mutation supported a mechanistic link between PCSK9-associated cholesterol handling and a growth-promoting metabolic program, consistent with functional data showing PCSK9 as therapeutic vulnerability in this genotype-defined subset [53]. Tumor-specific computational work in melanoma further connected PCSK9 expression to tumor immunity and predicted immunotherapy responsiveness, supporting the idea that PCSK9-linked pathways may shape immune escape in certain conditions [54]. Furthermore, a broader integrative analysis combining TCGA RNA-seq with GEO reported cancer type dependency to survival associations, which is shown to be beneficial in some cancers and adverse in others, reinforcing that clinical correlation of PCSK9 is not uniform across malignancies and needs to be further investigated based on the cancer type context [55]. Collectively, those studies provide hypothesis-generating evidence that PCSK9′s expression, prognostic value, and immune associations are highly context dependent and therefore require mechanistic validation in matched tumor models and clinically relevant immune environments.

**Table 1:** The studies of PCSK9 in association with cancer.

Cancer Type	Methods	Results	Mechanism	Ref.
Breast cancer	Human clinical cohort	Significant elevated circulating PCSK9 levels are measured in stage III breast cancer patients, in comparison to healthy individuals.	Not indicated	[24]
	In vivo study	Nanoliposomal anti-PCSK9 moderately improves breast cancer outcome by reducing tumor growth and extending lifespan in BALB/c mice inoculated with 4T1 breast carcinoma cells.	Systemic PCSK9 suppression improves LDLR activity and reduces pro-inflammatory signals, indirectly limiting tumor growth.	[56]
	In vitro study	PCSK9 inhibitor evolocumab, when combined with doxorubicin and trastuzumab, enhanced cell apoptosis and necrosis; inflammatory signaling-related molecules (MyD88-NLRP3-NF-κB-mTORC1 pathways) were suppressed.	PCSK9 inhibition attenuates chemo-induced cardiotoxicity by reducing MyD88/NLRP3 inflammasome activation, NF-κB signaling and downstream mTORC1 activation, while simultaneously improving anticancer efficacy.	[57]
	In vitro and in vivo studies	PCSK9 overexpression increases proliferation, sphere formation, and lung metastasis, while knockdown suppresses tumor growth and metastasis.	PCSK9 promotes LDLR degradation, altering membrane cholesterol and lipid-raft composition, thereby enhancing EGFR/HER3 signaling and a metastatic phenotype.	[58]
Head and Neck Squamous Cell Carcinoma (HNSCC)	Clinical analysis, in vitro and in vivo studies	Expression of PCSK9 is associated with decreased survival in human HNSCC; PCSK9 knockdown decreases ALDH1A1, CD44, CD133, SOX2, and Bmi1; reduces spheroid formation; increases CD8^+^ T-cell infiltration in vivo; and improves response to PD-1 blockade.	PCSK9 maintained a stem-like phenotype and restricted antitumor immunity by suppressing antigen presentation and T-cell infiltration.	[59]
	In silico and in vitro studies	In silico analysis suggests an association of PCSK9 with HNSCC progression. Yet, genetic deletion and pharmacologic inhibition of PCSK9 produce minimal changes in cell growth, spheroid formation, apoptosis, and invasion.	PCSK9 is considered a passenger gene in HNSCC, with no clear tumor-promoting mechanism identified.	[60]
Hepatocellular Carcinoma (HCC)	Clinical analysis, in vitro and in vivo studies	PCSK9 promoted resistance to sorafenib by activating AKT-s473 phosphorylation.	PCSK9 is involved in AKT activation signaling through the degradation of PTEN due to the palmitoylation of PCSK9.	[25]
	Clinical analysis, in vitro and in vivo studies	High PCSK9 correlated with poor prognosis; knockdown induced apoptosis and inhibited tumor growth.	PCSK9 suppressed mitochondrial apoptosis by regulating Bcl-2/Bax and caspase-9/3 activation.	[61]
Gastric cancer	Clinical analysis, in vitro and in vivo studies	PCSK9 overexpression in gastric cancer tissues was correlated with tumor progression and poor survival.	By upregulating HSP70, PCSK9 activates the MAPK signaling pathway and thus contributes to metastasis and the suppression of apoptosis in gastric cancer.	[62]
Lung cancer	In vitro study	PCSK9 siRNA induces apoptosis of A549 human lung adenocarcinoma cells and mitochondrial dysfunction.	The apoptotic effect of PCSK9 inhibition in lung cancer cells was associated with perturbation of mitochondrial membrane via Bax/Bcl-2 regulation and endoplasmic reticulum stress.	[63]
	Clinical and in vivo studies	High PCSK9 expression is associated with hindered effectiveness of anti-PD-1 immunotherapy, and the combination of PCSK9 inhibitor with anti-CD137 agonist retard tumor growth in Lewis lung carcinoma (LLC) mice model.	The antitumor effect of the PCSK9 inhibitor in combination with anti-CD137 was associated with the recruitment of CD8^+^ and GzmB+ CD8^+^ T cells, as well as the depletion of Tregs.	[64]
Colorectal cancer	Clinical analysis, in vitro and in vivo studies	Overexpression of PCSK9 is associated with poor survival in APC/KRAS-mutant CRC patients, and its depletion suppresses tumor growth in vitro and in vivo.	PCSK9 promotes APC/KRAS-mutant CRC via GGPP-KRAS/MEK/ERK pathway.	[53]
	In vitro and in vivo studies	PCSK9 inhibition increases CD8^+^ T-cell infiltration, reduces Treg accumulation, and sensitizes tumors to PD-1 blockade.	PCSK9 modulates the tumor immune microenvironment, limiting antigen presentation and cytotoxic T-cell recruitment.	[65]
Melanoma	In vitro and in vivo studies	PCSK9 and its gain-of-function variant (D374Y) increase melanoma cell proliferation, migration, and tumor growth in vivo. PCSK9-high tumors accumulate more cholesterol and display transcriptional signatures of T-cell dysfunction. PCSK9-network genes predict poor prognosis and associate with reduced response to immune checkpoint blockade.	PCSK9 enhances LDLR-dependent cholesterol uptake, drives melanoma progression, and induces an immune-dysfunction program that suppresses effective antitumor T-cell activity, thereby promoting resistance to immune checkpoint therapy.	[54]
	In vivo study	PCSK9 deficiency reduces liver metastatic burden and increases apoptotic signaling within metastatic lesions in Pcsk9^−^/^−^ mice.	Loss of PCSK9 lowers circulating LDL-cholesterol and enhances TNFα-mediated apoptosis, thereby limiting metastatic colonization in the liver.	[66]
Glioblastoma	In vitro study	PCSK9 knockdown induces apoptosis and mitochondrial dysfunction in U251 glioma cells, whereas PCSK9 overexpression supports cell survival.	PCSK9 modulates mitochondrial apoptosis through Bcl-2/Bax balance and caspase-9/3 activation, thereby controlling glioma cell viability.	[54]
	Clinical study	Evolocumab penetrates tumor tissue and increases surface MHC-I expression in resected glioblastoma samples.	PCSK9 inhibition restores antigen presentation by preventing lysosomal degradation of MHC-I, thereby enhancing tumor immunogenicity.	[67]
Pancreatic ductal adenocarcinoma (PDAC)	In vitro and in vivo studies	PCSK9 dictates organ tropism of PDAC metastasis—high PCSK9 directs metastasis toward the liver, whereas PCSK9 suppression shifts tropism toward the lung.	PCSK9 regulates LDL uptake and modifies cholesterol-intermediate pools that program organ-specific metastatic seeding.	[68]
Prostate cancer	In vitro study	PCSK9 knockdown reduces ionizing radiation-induced cell damage of PCa cell lines.	PCSK9 modulates radiation response by regulating the mitochondrial apoptosis pathway, which is linked to altered cytochrome c release, caspase-3 activation, and Bax/Bcl-2 balance.	[69]
	Patient tissue analysis, in vitro and in vivo studies	PCSK9 is detectable in patient tissue samples; its suppression reduces motility and colony formation of prostate cancer cells and attenuates recurrence in metastatic castration-resistant prostate cancer models in vivo.	PCSK9 supports castration-resistant tumor fitness through the PCSK9-LDLR/cholesterol axis, by regulating LDLR availability and cholesterol homeostasis.	[70,71]
Esophageal squamous cell carcinoma (ESCC)	Clinical analysis and in vitro study	PCSK9 is upregulated in ESCC, associated with poor prognosis; gain- and loss-of-function show functional effect in promoting proliferation, migration, and invasion.	PCSK9 promotes EMT via CCL25 secretion.	[72]
	Clinical cohort study	Higher serum anti-PCSK9-Ab is associated with better post-operative prognosis, and its antigen is detectable in ESCC tissue.	Not indicated.	[73]

## 5. Mechanisms Related to the Involvement of PCSK9 in Cancer

**Figure 1:**
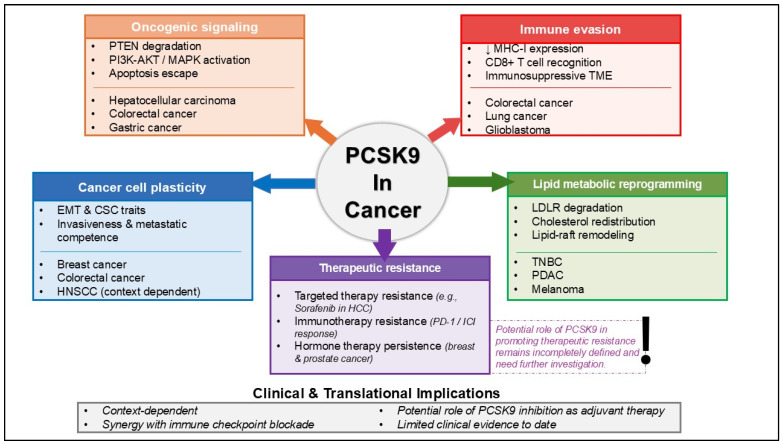
Overview of PCSK9 functions and proposed mechanisms in cancer, including therapeutic resistance.

PCSK9 has been extensively associated with cancer through multiple interconnected biological processes. An overview of the major biological mechanisms by which PCSK9 contributes to cancer progression and therapeutic resistance is summarized schematically in Figure 1. In addition, the key upstream regulators and downstream mediators implicated in PCSK9 signaling across cancer are presented in Table 2. Upstream mediators include transcriptional and post-translational regulators of PCSK9, whereas downstream mediators include molecular targets and cellular programs influenced by PCSK9 that shape tumor behavior. These mediators span tumor-intrinsic signaling pathways, immune-regulatory mechanisms, and lipid metabolic processes within the tumor microenvironment.

### 5.1. PCSK9-Driven Oncogenic Signaling

A central mechanism linking PCSK9 to tumor progression is its ability to reprogram intracellular signaling pathways. In hepatocellular carcinoma, PCSK9 binds PTEN and facilitates its lysosomal degradation in a palmitoylation-dependent manner, resulting in sustained activation of the PI3K–AKT pathway and diminished sensitivity to sorafenib [25]. This mechanistic observation is consistent with clinical associations between elevated PCSK9 expression and poorer survival in hepatocellular carcinoma [61], as well as experimental evidence showing that PCSK9 suppression restores mitochondrial apoptosis through the Bax/Bcl-2/caspase-9/3 axis [63].

Furthermore, PCSK9 amplifies oncogenic potential in APC/KRAS-mutant tumors through the KRAS–MEK–ERK cascade in colorectal cancer [53]. In gastric cancer, PCSK9 upregulates HSP70, enabling MAPK activation and protecting cells from apoptosis [62]. Breast cancer models further demonstrate that PCSK9 destabilizes LDLR and disrupts membrane lipid-raft composition, which enhances EGFR/HER3 signaling and supports metastatic behavior [58]. Similar pro-survival effects have been described in lung adenocarcinoma and glioma, where PCSK9 silencing leads to mitochondrial dysfunction and increased apoptosis [63,76]. Collectively, these results demonstrate that PCSK9 contributes to malignant progression by linking lipid-associated processes to the stability and activity of oncogenic signaling networks.

### 5.2. PCSK9 and Cancer Cell Plasticity: EMT and Stemness

PCSK9 influences cellular state transitions which contribute to the metastasis progression and therapeutic resistance. In colorectal cancer, PCSK9 promotes epithelial–mesenchymal transition (EMT) by coordinating PI3K–AKT activation with macrophage polarization, thereby supporting migratory and invasive properties [23].

A strong association between PCSK9 and stemness programs has been observed in head and neck squamous cell carcinoma (HNSCC) [59]. High PCSK9 expression coincides with increased levels of CSC-related markers, including BMI1, ALDH1A1, CD44, CD133, and SOX2, and its expression is further elevated under 3D culture conditions enriched for stem-like cells [59]. Spheroid formation in 3D culture was attenuated after PCSK9 downregulation. Moreover, by treating the cells with the PCSK9 inhibitor, several stemness markers, including CD44 and BMI1, were significantly reduced compared to non-treated cells. These results indicate the possibility that PCSK9 influences a stem-like phenotype in HNSCC [59]. However, another study reported that PCSK9 has minimal pathological influence in HNSCC and proposed PCSK9 as a passenger gene [60]. Notably, this study explicitly discussed the discrepancy with Yang et al. [59] and suggested that differences in experimental systems, including cell line selection and the use of human or mouse tumor models, may underlie the opposing results [60]. Together, these findings indicate that the contribution of PCSK9 to stemness in HNSCC may be context dependent and warrants further validation in clinically annotated cohorts and diverse model systems.

Other studies reinforce the relevance of this mechanism. In breast cancer, PCSK9 contributes to proliferation, sphere formation, and lung metastasis, and its suppression restricts these phenotypes [58]. Furthermore, melanoma models also reveal PCSK9 roles in facilitating proliferative and migratory behavior [54]. These observations collectively indicate that PCSK9 supports tumor progression by stabilizing phenotypic states associated with invasiveness, self-renewal, and metastatic competence.

### 5.3. PCSK9 and Immune Evasion

Many studies also demonstrated that PCSK9 affects cancer cells by regulating the immune system. PCSK9 evades the immune system by helping cancer cells escape recognition by cytotoxic T lymphocytes. This was supported by the reduction in cell surface major histocompatibility complex class I (MHC I) on the cell surface [20]. It was previously reported that cholesterol levels influence MHC I recycling [77]. Interestingly, the study [20] suggested that PCSK9 affects MHC I independently of its cholesterol-regulating function and that this effect may not be fully explained by cholesterol-dependent mechanisms [29], thereby preventing CD8^+^ T cells from recognizing and killing tumor cells.

In addition to its effect on antigen presentation, PCSK9 also promoted the infiltration of regulatory T cells (Treg) while limiting CD8+ T cell infiltration into the tumor, thus affecting the tumor microenvironment [65]. The study points out that in CRC models, PD-1 immune checkpoint inhibitors (ICI) in combination with anti-PCSK9 antibody exhibited enhanced antitumor effects. It is achieved through a synergistic effect of a PCSK9 antibody, which diminishes the increased expression of PCSK9, LDLR, TGF-β, and CD36 compared to the PD-1 inhibitor alone. Similarly, in lung cancer models, the combination of a PCSK9 inhibitor with an anti-CD137 agonist increases recruitment of CD8^+^ and granzyme-B^+^ cytotoxic T cells [64]. Glioblastoma tissue analyses further show that evolocumab penetrates tumor lesions and increases surface MHC-I expression [67]. More recently, Sun et al. [78] emphasized PCSK9 as a key immunomodulatory target in cancer, proposing its inhibition as a general strategy to enhance immune checkpoint therapy. This work synthesizes mechanistic and translational evidence supporting PCSK9-mediated regulation of antigen presentation and antitumor T-cell activity as a central mechanism underlying improved immunotherapeutic responses. Across these settings, PCSK9 functions as an immune-evasion regulator, limiting antigen presentation and restricting effector T-cell entry into tumors.

### 5.4. PCSK9-Mediated Lipid Reprogramming

Across different cancer systems, PCSK9 plays a broader metabolic role than initially recognized. Rather than acting solely as a regulator of LDLR abundance, PCSK9 influences how tumor cells distribute cholesterol and organize their plasma-membrane architecture [79]. Previous studies in hepatic models demonstrate that PCSK9 directs LDLR toward lysosomal degradation instead of recycling, resulting in a sustained reduction in surface LDLR and altering cholesterol availability within the cell [12,80]. Subsequent work showing that PCSK9 can also degrade VLDLR and ApoER2 further expands its impact on lipid uptake and receptor homeostasis [81].

These trafficking characteristics have clear implications for cancer biology. In triple-negative breast cancer, PCSK9-driven depletion of LDLR modifies membrane cholesterol composition and disrupts lipid-raft organization, changes that facilitate enhanced EGFR and HER3 signaling [58]. This shift favors proliferative, spheroid-forming, and metastatic phenotypes in experimental models. Melanoma studies similarly report that elevated PCSK9 leads to increased intracellular cholesterol accumulation and transcriptional programs associated with impaired T-cell activity and attenuated responses to immune checkpoint blockade, linking sterol metabolism to immune evasion [54].

Findings in PDAC further support the role for PCSK9 in cancer through this metabolic pathway [68]. Through its effects on LDL-cholesterol import and the production of sterol intermediates, PCSK9 influences the tendency of PDAC cells to metastasize to the liver versus the lung. Manipulating PCSK9 expression is sufficient to alter this organotropism in vivo, emphasizing its contribution to the metabolic conditions that support metastatic outgrowth in distinct tissues. Complementary insights from cardiovascular and hepatic research similarly suggest that PCSK9 overexpression can disrupt sterol balance in multiple cellular compartments and interact with inflammatory and metabolic pathways [79,82].

Overall, these observations support a model in which PCSK9 functions as a key coordinator of lipid metabolic dynamics in cancer. Through its effects on LDLR-family turnover and cholesterol flux, PCSK9 shapes lipid-raft integrity, modulates growth-factor and immune signaling, and helps define the metabolic landscape that enables tumor progression and metastatic behavior.

### 5.5. PCSK9, Endothelial Function, and Potential Tumor Angiogenesis

Beyond its tumor-intrinsic and immune-related functions, PCSK9 has been implicated in the regulation of endothelial function and angiogenesis in a context-dependent manner. In endothelial models, recombinant PCSK9 has been shown to enhance tube formation, consistent with pro-angiogenic activity under certain experimental conditions [83]. In contrast, studies in diabetic or high-glucose vascular contexts suggest that PCSK9 can impair angiogenic signaling by promoting NEDD4-mediated ubiquitination and degradation of VEGFR2, thereby reducing endothelial angiogenic capacity [84]. Additional work in hypoxia-related endothelial injury models further supports context-dependent effects of PCSK9 on angiogenic responses, as PCSK9 inhibition was reported to enhance angiogenesis in vivo [85]. While these findings highlight a regulatory role for PCSK9 in vascular biology, their direct relevance to tumor angiogenesis remains uncertain, as most evidence to date derives from non-malignant endothelial models.

## 6. Genetic and Clinical Evidence

Although PCSK9 has been widely investigated in cancer biology, the development of PCSK9-targeted therapies for oncology remains limited compared with their extensive use in cardiovascular disease. Nevertheless, human genetic and clinical observations increasingly suggest that PCSK9 may hold therapeutic relevance in oncology. A Mendelian randomization analysis showed that by inhibiting PCSK9, the risk of overall and early-onset prostate cancer was significantly reduced, with odds ratios (OR) = 0.85 and 0.7, respectively [86]. This effect has been suggested to be associated with the regulation of lipoprotein(a).

Clinical biomarker studies further show that circulating PCSK9 levels are elevated in several malignancies, including stage III breast cancer and hepatocellular carcinoma, where higher plasma or tissue expression correlates with aggressive disease features [24,87]. Complementary transcriptomic analyses across large patient cohorts reveal that PCSK9 is upregulated in multiple tumor types and displays context-dependent prognostic associations, often predicting poorer outcomes in colorectal, pancreatic, renal cancer, and melanoma [55]. These observations indicate that the contribution of PCSK9 to cancer biology varies across tumor types, likely reflecting differences in tissue-specific lipid metabolism, oncogenic signaling, and immune environment. Several clinical studies have also started to investigate targeting PCSK9 in combating cancer disease, as briefly summarized in a review by Oza & Kashfi [7]. Most clinical trials on it are targeting metastatic non-small cell lung cancer (NSCLC), and others are targeting high-grade glioma/glioblastoma and metastatic pancreatic cancer. One of them, NCT05128539, also focuses on testing the safety and early efficacy of anti-PCSK9 with toripalimab (a PD-1 inhibitor) on various advanced cancers of all types [88]. These emerging data, together with mechanistic insights linking PCSK9 to cholesterol handling, receptor signaling, and immune modulation, provide a coherent rationale for exploring PCSK9 inhibition as a complementary approach in cancer therapy. Accordingly, Table 3 summarizes currently available PCSK9-targeting drugs and experimental modalities, along with their proposed relevance to cancer treatment.

## 7. Contribution of PCSK9 in the Progression of Anticancer Drug Resistance

The mechanisms by which PCSK9 contributes to cancer progression, including apoptosis, immune system evasion, cancer stemness, and others, have been broadly associated with anticancer drug resistance. As illustrated in Figure 1, these PCSK9-regulated mechanisms converge to promote resistance to targeted therapy, immunotherapy, and hormone-based treatments. These observations suggest a role for PCSK9 in chemoresistance. However, direct evidence demonstrating how PCSK9 could contribute to cancer resistance remains limited. A study conducted by Sun et al. [25] indicates that PCSK9 is upregulated in HCC and has been shown to promote sorafenib resistance both in vitro and in vivo. Sorafenib, a multikinase inhibitor, is currently used as an effective first-line therapy for late-stage HCC. Unfortunately, the emergence of this drug resistance has become a significant problem. Mechanistically, S-palmitoylated PCSK9 binds to the tumor suppressor PTEN and accelerates its lysosomal degradation, leading to persistent activation of the PI3K/AKT pathway and reduced sensitivity to sorafenib. Blocking PCSK9 or inhibiting its palmitoylation restores PTEN levels and partially reverses resistance, indicating a tumor-intrinsic role for PCSK9 in kinase-inhibitor insensitivity.

Beyond kinase inhibitor resistance, current reports implicate PCSK9 in resistance to immune checkpoint blockade. By binding to MHC class I molecules and directing them toward lysosomal degradation, PCSK9 reduces MHC-I surface levels and weakens antigen presentation to cytotoxic T cells [20]. Studies using syngeneic colon cancer models have shown that PCSK9 inhibition enhances intratumoral CD8^+^ T-cell infiltration and improves the efficacy of anti-PD-1 therapy [65,92]. Similar findings have recently been reported in pancreatic ductal adenocarcinoma (PDAC). Lao et al. [93] demonstrate that SREBP1-driven upregulation of PCSK9 contributes to an immunosuppressive microenvironment by altering PD-L1 trafficking, leading to diminished responses to PD-1 blockade. In these models, the combination of PCSK9-neutralizing antibodies with PD-1 inhibitors resulted in substantially stronger antitumor activity than PD-1 monotherapy alone. Another study in microsatellite-stable colorectal cancer (MSS-CRC) further supports the role of PCSK9 in shaping immunotherapy response. Wang et al. [75] reported that methionine restriction suppresses PCSK9 expression and enhances the antitumor effect of PD-1 blockade, in part through increased MHC-I availability. PCSK9 has also been targeted more directly in preclinical systems. A nanoCRISPR platform designed to simultaneously disrupt PCSK9 and PD-L1 significantly increased tumor immunogenicity and improved immune-mediated tumor control [94]. Likewise, PCSK9-directed cVLP vaccines have been shown to augment the effects of HER2-targeted vaccination in HER2-positive mammary carcinoma models [95]. Together, these findings support a broader role for PCSK9 in modulating tumor–immune interactions that influence susceptibility to immunotherapy.

PCSK9-driven resistance is not limited to immune-based therapies. In hormone-dependent and castration-resistant tumors, modulation of the PCSK9–LDLR axis has been linked to tumor persistence and recurrence. In breast cancer, pseurotin A reduces PCSK9 secretion and interferes with PCSK9–LDLR interaction, leading to decreased tumor growth and recurrence [91]. Similar mechanisms have been described in metastatic castration-resistant prostate cancer (mCRPC), where PCSK9-targeting compounds, including pseurotin A and (−)-oleuropein, suppress PCSK9–LDLR binding and inhibit metastatic progression [71,96]. These studies suggest that cholesterol-transport pathways regulated by PCSK9 can support survival under endocrine therapies and contribute to resistance and relapse.

Overall, although the number of direct studies remains limited, growing evidence indicates that PCSK9 can influence sensitivity to sorafenib, immune checkpoint inhibitors, cellular immunotherapy, and hormone-based treatments. PCSK9 exerts its effects through tumor-intrinsic mechanisms, such as PTEN loss and enhanced survival signaling, together with immune-modulatory processes that diminish antigen presentation and alter PD-L1 regulation. Further mechanistic studies and early-phase clinical trials will be needed to determine the therapeutic value of combining PCSK9 inhibition with existing anticancer treatments and to identify the patient populations most likely to benefit from such strategies.

## 8. Conclusions and Future Directions

PCSK9 has been well known as a promising target for reducing the risk of cardiovascular disease by lowering plasma LDL-C levels. However, the function of PCSK9 is beyond that, as extensive studies have reported the overexpression of PCSK9 in multiple types of cancer and its contribution to cancer progression. These cancer-related activities have raised interest in whether PCSK9 inhibition could offer therapeutic benefit beyond cardiovascular indications. Furthermore, PCSK9 has also been shown to influence angiogenic responses in endothelial systems, suggesting potential effects on tumor angiogenesis. However, their direct relevance remains unexplored; thus, any vascular effects of PCSK9 inhibition in oncology should currently be considered exploratory. At present, direct evidence supporting PCSK9 inhibition as a consistent anti-angiogenic strategy in cancer is limited, and any vascular effects are likely to be tumor-type and microenvironment dependent. Current evidence in cancer-related primarily supports PCSK9 inhibition as a potential immunomodulatory strategy, especially in combination with immune checkpoint inhibitors, whereas clear clinical efficacy evidence is still limited.

Furthermore, although direct studies remain limited, emerging work across hepatocellular carcinoma, pancreatic cancer, colorectal cancer, and hormone-related tumors suggests that PCSK9 may influence responses to sorafenib, immune checkpoint blockade, and endocrine-based therapies. These findings point to a potential role for PCSK9 as both a biomarker of therapy response and a complementary target to enhance existing anticancer strategies. However, the precise mechanisms by which PCSK9 contributes to drug resistance, particularly within the tumor microenvironment, remain unresolved.

Future research should focus on clarifying the context-specific roles of PCSK9 in resistance pathways, identifying reliable biomarkers of PCSK9 activity, and determining which tumor types are most likely to benefit from PCSK9-targeted approaches. Preclinical investigation of combination strategies—such as PCSK9 inhibition alongside kinase inhibitors, PD-1 blockade, or metabolic interventions—will be essential, followed by early-phase clinical trials to evaluate safety, dosing, and therapeutic efficacy. A deeper mechanistic understanding will be crucial to establish whether PCSK9 inhibition can meaningfully reverse or prevent anticancer drug resistance.

## Figures and Tables

**Table 2:** Molecular and cellular mediators associated with PCSK9 signaling in cancer.

Functional Axis/Mechanisms	Mediators	Roles	Mechanistic Association with PCSK9	Ref.
Antigen presentation/immune evasion	MHC class I (MHC-I/HLA-I)	Downstream molecular mediator	PCSK9 associates with MHC-I and promotes lysosomal degradation, reducing tumor cell surface MHC-I and antigen presentation.	[20]
Antitumor immunity	CD8^+^ T cells	Downstream cellular consequence	PCSK9 inhibition or knockdown increases intratumoral CD8^+^ T cell infiltration and improves antitumor activity, which is often enhanced with anti-PD-1.	[20]
Immunotherapy sensitization	Regulatory T cells (Tregs)	Downstream cellular consequence	PCSK9 inhibition enhances the antitumor effect of PD-1 blockade in CRC models and is reported to promote CD8^+^ infiltration with Treg exclusion.	[65]
LDLR-axis phenotypes	LDLR	Downstream molecular mediator	Canonical PCSK9 target; in cancer cells, PCSK9-driven LDLR turnover reshapes membrane cholesterol and lipid-raft organization, which can enhance receptor-dependent signaling and downstream malignant behaviors in a tumor type-dependent manner.	[58]
Growth factor signaling	EGFR & HER3	Downstream signaling effector	PCSK9-LDLR-dependent plasma membrane cholesterol or lipid-raft remodeling is associated with increased EGFR and HER3 activation and malignant phenotypes in TNBC models.	[58]
Migration	CCL25	Downstream effector	PCSK9 promotes ESCC proliferation/migration by facilitating CCL25 secretion.	[72]
Metastasis/apoptosis resistance	HSP70	Downstream effector	PCSK9 upregulates HSP70 and facilitates MAPK signaling, linked to apoptosis suppression and metastasis in gastric cancer models.	[62]
Drug resistance	ZDHHC16	Upstream regulator	ZDHHC16 mediates S-palmitoylation of PCSK9, increasing PCSK9-PTEN interaction and contributing to sorafenib resistance in HCC models.	[25]
Drug resistance	PTEN	Downstream effector	Palmitoylated PCSK9 promotes lysosomal PTEN degradation → AKT activation → reduced sorafenib sensitivity.	[25]
Tumor suppression	GSTP1	Downstream interacting partner	PCSK9 interacts with GSTP1 and suppresses JNK signaling; reported tumor-suppressive effect in an HCC study.	[74]
Transcriptional regulation	DNMT1 & SIRT6	Upstream epigenetic regulator	Methionine/SAM axis promotes PCSK9 transcription via DNMT1-dependent DNA methylation in colorectal cancer; reduced SIRT6 is linked to increased PCSK9 transcription within the same CRC methionine-PCSK9 axis.	[75]

**Table 3:** PCSK9-targeting drugs and modalities with potential relevance in cancer therapy.

Modality/Drug	Target and Mechanism of Action	Relevance in Cancer	Oncology Clinical Evaluation	Ref.
Evolocumab (mAb)	Neutralizes circulating PCSK9, preventing PCSK9-mediated LDLR degradation and enhancing LDLR recycling.	In preclinical tumor models, PCSK9 inhibition increases tumor cell surface MHC-I by preventing lysosomal degradation, improves antigen presentation, and synergizes with anti-PD-1 therapy.	Phase II: Registered oncology trial of evolocumab combined with Nivolumab PD-1 blockade in metastatic renal cell carcinoma.	[20,89]
Alirocumab (mAb)	Neutralizes circulating PCSK9, preventing PCSK9-mediated LDLR degradation and enhancing LDLR recycling.	Mechanistically expected to enhance antitumor immunity through PCSK9 inhibition based on the PCSK9-MHC-I axis; supports combination strategies with immune checkpoint blockade.	Phase II: Registered oncology trial of alirocumab combined with Cemiplimab PD-1 blockade in metastatic non-small cell lung cancer after ICI progression.	[90]
JS002 (mAb)	Neutralizes circulating PCSK9, preventing PCSK9-mediated LDLR degradation and enhancing LDLR recycling.	Evaluated clinically in combination with a PD-1 inhibitor for safety and preliminary efficacy in advanced solid tumors.	Phase I: JS002 in combination with toripalimab in advanced solid tumors (safety and preliminary efficacy). The trial has been terminated considering the prespecified efficacy criteria were not met.	[88]
Inclisiran (siRNA)	Hepatocyte-targeted siRNA that suppresses hepatic PCSK9 synthesis.	Provides sustained systemic PCSK9 suppression; proposed to relieve PCSK9-mediated immune evasion and support immunotherapy efficacy.	No established oncology trials identified (approved for lipid lowering; oncology relevance is currently rationale-based).	[15]
PCSK9 vaccine (experimental)	Active immunization to induce endogenous anti-PCSK9 antibodies enabling long-term PCSK9 neutralization.	Potential strategy for durable PCSK9 suppression to support long-term cancer immunotherapy combinations.	Preclinical.	[56]
Small-molecule/natural product PCSK9-axis inhibitors (e.g., pseurotin A)	Suppress PCSK9 expression and/or disrupt PCSK9-LDLR interaction.	Reduce migration, clonogenic growth, and recurrence in prostate and breast cancer models; useful for probing PCSK9-LDLR axis draggability.	Preclinical.	[70,71,91]
Genetic depletion (siRNA/CRISPR)	Direct suppression or deletion of PCSK9 in tumor or host cells.	Demonstrates tumor-intrinsic and host-mediated roles of PCSK9 in immune evasion, metastasis, and therapy resistance.	Not applicable (mechanistic validation).	[20]

## Data Availability

No new data were created or analyzed in this study.

## Abbreviations

AKT, protein kinase B (serine/threonine kinase); ALDH1A1, aldehyde dehydrogenase 1 family member A1; APC, adenomatous polyposis coli; ApoER2, apolipoprotein E receptor 2 (also known as LRP8); ATP, adenosine triphosphate; BAX, BCL2-associated X protein; BCL-2, B-cell lymphoma 2; BMI1, B lymphoma Mo-MLV insertion region 1 homolog; cBioPortal, cBioPortal for Cancer Genomics; cDNA, complementary DNA; CD8^+^, cluster of differentiation 8 positive (T cells); CD36, cluster of differentiation 36; COAD, colon adenocarcinoma (TCGA cohort); CRC, colorectal cancer; CRISPR, clustered regularly interspaced short palindromic repeats; CSC, cancer stem cell(s); CTLA-4, cytotoxic T-lymphocyte–associated protein 4; D374Y, aspartate-to-tyrosine substitution at position 374 (PCSK9 gain-of-function variant); DNA, deoxyribonucleic acid; DNMT1, DNA methyltransferase 1; EGFR, epidermal growth factor receptor; EMT, epithelial–mesenchymal transition; ERK, extracellular signal-regulated kinase; ESCC, esophageal squamous cell carcinoma; FADH2, flavin adenine dinucleotide (reduced form); FAO, fatty acid oxidation; FASN, fatty acid synthase; GEO, Gene Expression Omnibus; GGPP, geranylgeranyl pyrophosphate; GzmB, granzyme B; GSTP1, glutathione S-transferase pi 1; HCC, hepatocellular carcinoma; HER2, human epidermal growth factor receptor 2 (ERBB2); HER3, human epidermal growth factor receptor 3 (ERBB3); HLA-I, human leukocyte antigen class I; HNSCC, head and neck squamous cell carcinoma; HSP70, heat shock protein 70; ICI, immune checkpoint inhibitor(s); JNK, c-Jun N-terminal kinase; JS002, JS002 (anti-PCSK9 monoclonal antibody; drug code/name); KRAS, Kirsten rat sarcoma viral oncogene homolog; LDL, low-density lipoprotein; LDL-C, low-density lipoprotein cholesterol; LDLR, low-density lipoprotein receptor; LLC, Lewis lung carcinoma; LRP8, LDL receptor related protein 8 (ApoER2); MAPK, mitogen-activated protein kinase; mAb, monoclonal antibody; MHC-I, major histocompatibility complex class I; MHC-II, major histocompatibility complex class II; mCRPC, metastatic castration-resistant prostate cancer; MEK, mitogen-activated protein kinase; mTORC1, mechanistic target of rapamycin complex 1; MyD88, myeloid differentiation primary response 88; MSS-CRC, microsatellite-stable colorectal cancer; NADH, nicotinamide adenine dinucleotide (reduced form); NARC-1, neural apoptosis-regulated convertase 1 (another name for PCSK9); NEDD4, neural precursor cell expressed developmentally down-regulated protein 4 (E3 ubiquitin ligase); NF-κB, nuclear factor kappa B; NLRP3, NLR family pyrin domain containing 3; NSCLC, non-small cell lung cancer; OR, odds ratio; PCa, prostate cancer; PCSK9, proprotein convertase subtilisin/kexin type 9; PD-1, programmed cell death protein 1; PD-L1, programmed death-ligand 1; PDAC, pancreatic ductal adenocarcinoma; PI3K, phosphoinositide 3-kinase; PTEN, phosphatase and tensin homolog; READ, rectum adenocarcinoma (TCGA cohort); RNA-seq, RNA sequencing; SAM, S-adenosylmethionine; siRNA, small interfering RNA; SIRT6, sirtuin 6; SOX2, SRY-box transcription factor 2; SREBP, sterol regulatory element-binding protein; TCGA, The Cancer Genome Atlas; TGF-β, transforming growth factor beta; TNBC, triple-negative breast cancer; TNFα, tumor necrosis factor alpha; Treg/Tregs, regulatory T cell(s); VLDLR, very-low-density lipoprotein receptor; VEGFR2, vascular endothelial growth factor receptor 2; ZDHHC16, zinc finger DHHC-type palmitoyltransferase 16.

## References

[1] Kocarnik J.M., Compton K., Dean F.E., Fu W., Gaw B.L., Harvey J.D., Henrikson H.J., Lu D., Pennini A., Xu R. (2022). Cancer Incidence, Mortality, Years of Life Lost, Years Lived With Disability, and Disability-Adjusted Life Years for 29 Cancer Groups From 2010 to 2019 A Systematic Analysis for the Global Burden of Disease Study 2019. JAMA Oncol..

[2] Bukowski K., Kciuk M., Kontek R. (2020). Mechanisms of Multidrug Resistance in Cancer Chemotherapy. Int. J. Mol. Sci..

[3] Han J., Lim W., You D., Jeong Y., Kim S., Lee J.E., Shin T.H., Lee G., Park S. (2019). Chemoresistance in the Human Triple-Negative Breast Cancer Cell Line MDA-MB-231 Induced by Doxorubicin Gradient Is Associated with Epigenetic Alterations in Histone Deacetylase. J. Oncol..

[4] Abdulla N., Vincent C.T., Kaur M. (2021). Mechanistic Insights Delineating the Role of Cholesterol in Epithelial Mesenchymal Transition and Drug Resistance in Cancer. Front. Cell Dev. Biol..

[5] Prieto-Vila M., Takahashi R.U., Usuba W., Kohama I., Ochiya T. (2017). Drug Resistance Driven by Cancer Stem Cells and Their Niche. Int. J. Mol. Sci..

[6] Germain N., Dhayer M., Boileau M., Fovez Q., Kluza J., Marchetti P. (2020). Lipid Metabolism and Resistance to Anticancer Treatment. Biology.

[7] Oza P.P., Kashfi K. (2023). The Evolving Landscape of PCSK9 Inhibition in Cancer. Eur. J. Pharmacol..

[8] Abifadel M., Varret M., Rabès J.P., Allard D., Ouguerram K., Devillers M., Cruaud C., Benjannet S., Wickham L., Erlich D. (2003). Mutations in PCSK9 Cause Autosomal Dominant Hypercholesterolemia. Nat. Genet..

[9] Seidah N.G., Benjannet S., Wickham L., Marcinkiewicz J., Bélanger Jasmin S., Stifani S., Basak A., Prat A., Chrétien M. (2003). The Secretory Proprotein Convertase Neural Apoptosis-Regulated Convertase 1 (NARC-1): Liver Regeneration and Neuronal Differentiation. Proc. Natl. Acad. Sci. USA.

[10] Horton J.D., Cohen J.C., Hobbs H.H. (2007). Molecular Biology of PCSK9: Its Role in LDL Metabolism. Trends Biochem. Sci..

[11] Lagace T.A., Curtis D.E., Garuti R., McNutt M.C., Sahng W.P., Prather H.B., Anderson N.N., Ho Y.K., Hammer R.E., Horton J.D. (2006). Secreted PCSK9 Decreases the Number of LDL Receptors in Hepatocytes and in Livers of Parabiotic Mice. J. Clin. Investig..

[12] Maxwell K.N., Fisher E.A., Breslow J.L. (2005). Overexpression of PCSK9 Accelerates the Degradation of the LDLR in a Post-Endoplasmic Reticulum Compartment. Proc. Natl. Acad. Sci. USA.

[13] Robinson J.G., Nedergaard B.S., Rogers W.J., Fialkow J., Neutel J.M., Ramstad D., Somaratne R., Legg J.C., Nelson P., Scott R. (2014). Effect of Evolocumab or Ezetimibe Added to Moderate- Or High-Intensity Statin Therapy on LDL-C Lowering in Patients with Hypercholesterolemia: The LAPLACE-2 Randomized Clinical Trial. JAMA.

[14] Robinson J.G., Farnier M., Krempf M., Bergeron J., Luc G., Averna M., Stroes E.S., Langslet G., Raal F.J., El Shahawy M. (2015). Efficacy and Safety of Alirocumab in Reducing Lipids and Cardiovascular Events. N. Engl. J. Med..

[15] Ray K.K., Wright R.S., Kallend D., Koenig W., Leiter L.A., Raal F.J., Bisch J.A., Richardson T., Jaros M., Wijngaard P.L.J. (2020). Two Phase 3 Trials of Inclisiran in Patients with Elevated LDL Cholesterol. N. Engl. J. Med..

[16] Sabatine M.S., Giugliano R.P., Keech A.C., Honarpour N., Wiviott S.D., Murphy S.A., Kuder J.F., Wang H., Liu T., Wasserman S.M. (2017). Evolocumab and Clinical Outcomes in Patients with Cardiovascular Disease. N. Engl. J. Med..

[17] Schwartz G.G., Steg P.G., Szarek M., Bhatt D.L., Bittner V.A., Diaz R., Edelberg J.M., Goodman S.G., Hanotin C., Harrington R.A. (2018). Alirocumab and Cardiovascular Outcomes after Acute Coronary Syndrome. N. Engl. J. Med..

[18] Schmidt A.F., Carter J.-P.L., Pearce L.S., Wilkins J.T., Overington J.P., Hingorani A.D., Casas J.P. (2020). PCSK9 monoclonal antibodies for the primary and secondary prevention of cardiovascular disease. Cochrane Database Syst Rev..

[19] Bao X., Liang Y., Chang H., Cai T., Feng B., Gordon K., Zhu Y., Shi H., He Y., Xie L. (2024). Targeting Proprotein Convertase Subtilisin/Kexin Type 9 (PCSK9): From Bench to Bedside. Signal Transduct. Target. Ther..

[20] Liu X., Bao X., Hu M., Chang H., Jiao M., Cheng J., Xie L., Huang Q., Li F., Li C.Y. (2020). Inhibition of PCSK9 Potentiates Immune Checkpoint Therapy for Cancer. Nature.

[21] Bhattacharya A., Chowdhury A., Chaudhury K., Shukla P.C. (2021). Proprotein Convertase Subtilisin/Kexin Type 9 (PCSK9): A Potential Multifaceted Player in Cancer. Biochim. Biophys. Acta Rev. Cancer.

[22] Sun C., Zhu G., Shen C., Huang S., Li R., Li J., Ma Z., Wang Z. (2023). Identification and Validation of PCSK9 as a Prognostic and Immune-Related Influencing Factor in Tumorigenesis: A Pan-Cancer Analysis. Front. Oncol..

[23] Wang L., Li S., Luo H., Lu Q., Yu S. (2022). PCSK9 Promotes the Progression and Metastasis of Colon Cancer Cells through Regulation of EMT and PI3K/AKT Signaling in Tumor Cells and Phenotypic Polarization of Macrophages. J. Exp. Clin. Cancer Res..

[24] Wong Chong E., Joncas F.H., Seidah N.G., Calon F., Diorio C., Gangloff A. (2022). Circulating Levels of PCSK9, ANGPTL3 and Lp(a) in Stage III Breast Cancers. BMC Cancer.

[25] Sun Y., Zhang H., Meng J., Guo F., Ren D., Wu H., Jin X. (2022). S-Palmitoylation of PCSK9 Induces Sorafenib Resistance in Liver Cancer by Activating the PI3K/AKT Pathway. Cell Rep..

[26] Ferri N., Corsini A., Macchi C., Magni P., Ruscica M. (2016). Proprotein Convertase Subtilisin Kexin Type 9 and High-Density Lipoprotein Metabolism: Experimental Animal Models and Clinical Evidence. Transl. Res..

[27] Hummelgaard S., Vilstrup J.P., Gustafsen C., Glerup S., Weyer K. (2023). Targeting PCSK9 to Tackle Cardiovascular Disease. Pharmacol. Ther..

[28] Xia X.D., Peng Z.S., Gu H.M., Wang M., Wang G.Q., Zhang D.W. (2021). Regulation of PCSK9 Expression and Function: Mechanisms and Therapeutic Implications. Front. Cardiovasc. Med..

[29] Abifadel M., Rabès J.P., Devillers M., Munnich A., Erlich D., Junien C., Varret M., Boileau C. (2009). Mutations and Polymorphisms in the Proprotein Convertase Subtilisin Kexin 9 (PCSK9) Gene in Cholesterol Metabolism and Disease. Hum. Mutat..

[30] Cohen J.C., Boerwinkle E., Mosley T.H., Hobbs H.H. (2006). Sequence Variations in PCSK9, Low LDL, and Protection against Coronary Heart Disease. N. Engl. J. Med..

[31] Chen H., Chen X. (2023). PCSK9 Inhibitors for Acute Coronary Syndrome: The Era of Early Implementation. Front. Cardiovasc. Med..

[32] Lagace T.A. (2014). PCSK9 and LDLR Degradation: Regulatory Mechanisms in Circulation and in Cells. Curr. Opin. Lipidol..

[33] Raedler L.A. (2016). Praluent (Alirocumab): First PCSK9 Inhibitor Approved by the FDA for Hypercholesterolemia. Am. Health Drug Benefits.

[34] Fala L. (2016). Repatha (Evolocumab): Second PCSK9 Inhibitor Approved by the FDA for Patients with Familial Hypercholesterolemia. Am. Health Drug Benefits.

[35] Cicero A.F.G., Fogacci F., Zambon A., Toth P.P., Borghi C. (2022). Efficacy and Safety of Inclisiran a Newly Approved FDA Drug: A Systematic Review and Pooled Analysis of Available Clinical Studies. Am. Heart J. Plus Cardiol. Res. Pract..

[36] Santos C.R., Schulze A. (2012). Lipid Metabolism in Cancer. FEBS J..

[37] Snaebjornsson M.T., Janaki-Raman S., Schulze A. (2020). Greasing the Wheels of the Cancer Machine: The Role of Lipid Metabolism in Cancer. Cell Metab..

[38] Koundouros N., Poulogiannis G. (2020). Reprogramming of Fatty Acid Metabolism in Cancer. Br. J. Cancer.

[39] Griffiths B., Lewis C.A., Bensaad K., Ros S., Zhang Q., Ferber E.C., Konisti S., Peck B., Miess H., East P. (2013). Sterol Regulatory Element Binding Protein-Dependent Regulation of Lipid Synthesis Supports Cell Survival and Tumor Growth. Cancer Metab..

[40] Wen Y.A., Xiong X., Zaytseva Y.Y., Napier D.L., Vallee E., Li A.T., Wang C., Weiss H.L., Evers B.M., Gao T. (2018). Downregulation of SREBP Inhibits Tumor Growth and Initiation by Altering Cellular Metabolism in Colon Cancer Article. Cell Death Dis..

[41] Bensaad K., Favaro E., Lewis C.A., Peck B., Lord S., Collins J.M., Pinnick K.E., Wigfield S., Buffa F.M., Li J.L. (2014). Fatty Acid Uptake and Lipid Storage Induced by HIF-1α Contribute to Cell Growth and Survival after Hypoxia-Reoxygenation. Cell Rep..

[42] Jin Y., Tan Y., Wu J., Ren Z. (2023). Lipid Droplets: A Cellular Organelle Vital in Cancer Cells. Cell Death Discov..

[43] Simons K., Ikonen E. (1997). Functional Rafts in Cell Membranes. Nature.

[44] Huang B., Song B.-L., Xu C. (2020). Cholesterol Metabolism in Cancer: Mechanisms and Therapeutic Opportunities. Nat. Metab..

[45] Civita S., Ruglioni M., Mariangeli M., Barachini S., Salvadori T., Cristiani S., Carnicelli V., Petrini I., Nepita I., Castello M. (2025). Nanoscale Engagement of Programmed Death Ligand 1 (PD-L1) in Membrane Lipid Raft Domains of Cancer Cells. FEBS J..

[46] Pascual G., Avgustinova A., Mejetta S., Martín M., Castellanos A., Attolini C.S.O., Berenguer A., Prats N., Toll A., Hueto J.A. (2017). Targeting Metastasis-Initiating Cells through the Fatty Acid Receptor CD36. Nature.

[47] Peetla C., Vijayaraghavalu S., Labhasetwar V. (2013). Biophysics of Cell Membrane Lipids in Cancer Drug Resistance: Implications for Drug Transport and Drug Delivery with Nanoparticles. Adv. Drug Deliv. Rev..

[48] Li J., Dong L., Wei D., Wang X., Zhang S., Li H. (2014). Fatty Acid Synthase Mediates the Epithelial-Mesenchymal Transition of Breast Cancer Cells. Int. J. Biol. Sci..

[49] Li H., Feng Z., He M.L. (2020). Lipid Metabolism Alteration Contributes to and Maintains the Properties of Cancer Stem Cells. Theranostics.

[50] Liu H., Zhang Z., Song L., Gao J., Liu Y. (2022). Lipid Metabolism of Cancer Stem Cells (Review). Oncol. Lett..

[51] Cui C., Yan A., Huang S., Chen Y., Zhao J., Li C., Wang X., Yang J. (2025). PCSK9 Manipulates Lipid Metabolism and the Immune Microenvironment in Cancer. Onco Targets Ther..

[52] Wang H., Zhang X., Zhang Y., Shi T., Zhang Y., Song X., Liu B., Wang Y., Wei J. (2024). Targeting PCSK9 to Upregulate MHC-II on the Surface of Tumor Cells in Tumor Immunotherapy. BMC Cancer.

[53] Wong C.C., Wu J.L., Ji F., Kang W., Bian X., Chen H., Chan L.S., Luk S.T.Y., Tong S., Xu J. (2022). The Cholesterol Uptake Regulator PCSK9 Promotes and Is a Therapeutic Target in APC/KRAS-Mutant Colorectal Cancer. Nat. Commun..

[54] Gu Y., Lin X., Dong Y., Wood G., Seidah N.G., Werstuck G., Major P., Bonert M., Kapoor A., Tang D. (2023). PCSK9 Facilitates Melanoma Pathogenesis via a Network Regulating Tumor Immunity. J. Exp. Clin. Cancer Res..

[55] Ungvari Z., Menyhart O., Lehoczki A., Fekete M., Bianchini G., Győrffy B. (2025). PCSK9 Expression and Cancer Survival: A Prognostic Biomarker at the Intersection of Oncology and Geroscience. Geroscience.

[56] Momtazi-Borojeni A.A., Nik M.E., Jaafari M.R., Banach M., Sahebkar A. (2019). Effects of Immunization against PCSK9 in an Experimental Model of Breast Cancer. Arch. Med. Sci..

[57] Quagliariello V., Buccolo S., Iovine M., Paccone A., Bonelli A., Cavalcanti E., Rea D., De Laurentiis M., Botti G., Maurea N. (2021). PCSK9 Inhibitor Evolocumab to Increase Anticancer Activities and Reduce Cardiotoxicity during Doxorubicin and Trastuzumab, as Sequential Treatment, through MyD88/NF-KB/MTORC1 Pathways. J. Clin. Oncol..

[58] Li T., Wu R., Luo K.Q. (2025). PCSK9 Promotes the Malignancy of Triple-negative Breast Cancer Cells by Reducing Cholesterol Levels at the Plasma Membrane to Activate EGFR and HER3. Adv. Sci..

[59] Yang Q.C., Wang S., Liu Y.T., Song A., Wu Z.Z., Wan S.C., Li H.M., Sun Z.J. (2023). Targeting PCSK9 Reduces Cancer Cell Stemness and Enhances Antitumor Immunity in Head and Neck Cancer. iScience.

[60] Kim H.-J., Park D.-G., Choi S.-J., Cho S.-D. (2025). PCSK9 Is a Passenger Gene in Head and Neck Cancer with Minimal Pathological Influence. Arch. Oral Biol..

[61] Zhang S.Z., Zhu X.D., Feng L.H., Li X.L., Liu X.F., Sun H.C., Tang Z.Y. (2021). PCSK9 Promotes Tumor Growth by Inhibiting Tumor Cell Apoptosis in Hepatocellular Carcinoma. Exp. Hematol. Oncol..

[62] Xu B., Li S., Fang Y., Zou Y., Song D., Zhang S., Cai Y. (2021). Proprotein Convertase Subtilisin/Kexin Type 9 Promotes Gastric Cancer Metastasis and Suppresses Apoptosis by Facilitating MAPK Signaling Pathway Through HSP70 Up-Regulation. Front. Oncol..

[63] Xu X., Cui Y., Cao L., Zhang Y., Yin Y., Hu X. (2017). PCSK9 Regulates Apoptosis in Human Lung Adenocarcinoma A549 Cells via Endoplasmic Reticulum Stress and Mitochondrial Signaling Pathways. Exp. Ther. Med..

[64] Gao X., Yi L., Jiang C., Li S., Wang X., Yang B., Li W., Che N., Wang J., Zhang H. (2023). PCSK9 Regulates the Efficacy of Immune Checkpoint Therapy in Lung Cancer. Front. Immunol..

[65] Wang R., Liu H., He P., An D., Guo X., Zhang X., Feng M. (2022). Inhibition of PCSK9 Enhances the Antitumor Effect of PD-1 Inhibitor in Colorectal Cancer by Promoting the Infiltration of CD8+ T Cells and the Exclusion of Treg Cells. Front. Immunol..

[66] Sun X., Essalmani R., Day R., Khatib A.M., Seidah N.G., Prat A. (2012). Proprotein Convertase Subtilisin/Kexin Type 9 Deficiency Reduces Melanoma Metastasis in Liver. Neoplasia.

[67] Singh K., Foster M.W., Violette M.J., Corcoran A.M., Hotchkiss K.M., Railton C.O., Blandford E.E., Blethen K.E., Thomas E.L., McIntosh W.C. (2025). A Surgical Window of Opportunity Trial Evaluating the Effect of the PCSK9 Inhibitor Evolocumab on Tumoral MHC-I Expression and CD8+ Infiltration in Glioma. Sci. Rep..

[68] Rademaker G., Hernandez G.A., Seo Y., Dahal S., Miller-Phillips L., Li A.L., Peng X.L., Luan C., Qiu L., Liegeois M.A. (2025). PCSK9 Drives Sterol-Dependent Metastatic Organ Choice in Pancreatic Cancer. Nature.

[69] Gan S.S., Ye J.Q., Wang L., Qu F.J., Chu C.M., Tian Y.J., Yang W., Cui X.G. (2017). Inhibition of PCSK9 Protects against Radiationinduced Damage of Prostate Cancer Cells. Onco Targets Ther..

[70] Abdelwahed K.S., Siddique A.B., Qusa M.H., King J.A., Souid S., Abd Elmageed Z.Y., El Sayed K.A. (2021). PCSK9 Axis-Targeting Pseurotin A as a Novel Prostate Cancer Recurrence Suppressor Lead. ACS Pharmacol. Transl. Sci..

[71] Abdelwahed K.S., Siddique A.B., Ebrahim H.Y., Qusa M.H., Mudhish E.A., Rad A.H., Zerfaoui M., Abd Elmageed Z.Y., El Sayed K.A. (2023). Pseurotin A Validation as a Metastatic Castration-Resistant Prostate Cancer Recurrence-Suppressing Lead via PCSK9-LDLR Axis Modulation. Mar. Drugs.

[72] Wang H., Guo Q., Wang M., Liu C., Tian Z. (2023). PCSK9 Promotes Tumor Cell Proliferation and Migration by Facilitating CCL25 Secretion in Esophageal Squamous Cell Carcinoma. Oncol. Lett..

[73] Ito M., Hiwasa T., Oshima Y., Yajima S., Suzuki T., Nanami T., Sumazaki M., Shiratori F., Funahashi K., Li S.Y. (2021). Association of Serum Anti-PCSK9 Antibody Levels with Favorable Postoperative Prognosis in Esophageal Cancer. Front. Oncol..

[74] He M., Hu J., Fang T., Tang W., Lv B., Yang B., Xia J. (2022). Protein Convertase Subtilisin/Kexin Type 9 Inhibits Hepatocellular Carcinoma Growth by Interacting with GSTP1 and Suppressing the JNK Signaling Pathway. Cancer Biol. Med..

[75] Wang Q., Chen Z., Lu X., Lin H., Feng H., Weng N., Chen L., Liu M., Long L., Huang L. (2025). Methionine Metabolism Dictates PCSK9 Expression and Antitumor Potency of PD-1 Blockade in MSS Colorectal Cancer. Adv. Sci..

[76] Piao M.X., Bai J.W., Zhang P.F., Zhang Y.Z. (2015). PCSK9 Regulates Apoptosis in Human Neuroglioma U251 Cells via Mitochondrial Signaling Pathways. Int. J. Clin. Exp. Pathol..

[77] Naslavsky N., Weigert R., Donaldson J.G. (2004). Characterization of a Nonclathrin Endocytic Pathway: Membrane Cargo and Lipid Requirements. Mol. Biol. Cell.

[78] Sun S., Ma J., Zuo T., Shi J., Sun L., Meng C., Shu W., Yang Z., Yao H., Zhang Z. (2024). Inhibition of PCSK9: A Promising Enhancer for Anti-PD-1/PD-L1 Immunotherapy. Research.

[79] Seidah N.G., Prat A. (2021). The Multifaceted Biology of PCSK9. Endocr. Rev..

[80] Zhang D.W., Garuti R., Tang W.J., Cohen J.C., Hobbs H.H. (2008). Structural Requirements for PCSK9-Mediated Degradation of the Low-Density Lipoprotein Receptor. Proc. Natl. Acad. Sci. USA.

[81] Poirier S., Mayer G., Benjannet S., Bergeron E., Marcinkiewicz J., Nassoury N., Mayer H., Nimpf J., Prat A., Seidah N.G. (2008). The Proprotein Convertase PCSK9 Induces the Degradation of Low Density Lipoprotein Receptor (LDLR) and Its Closest Family Members VLDLR and ApoER2. J. Biol. Chem..

[82] Ajoolabady A., Pratico D., Mazidi M., Davies I.G., Lip G.Y.H., Seidah N., Libby P., Kroemer G., Ren J. (2025). PCSK9 in Metabolism and Diseases. Metabolism.

[83] Tsouka A., Koutsaliaris I., Tselepis A. (2025). Recombinant PCSK9 Enhances Angiogenesis in Endothelial Cells, While Lipoproteins Suppress Vegf-Induced Angiogenic Activity in Vitro. Atherosclerosis.

[84] Gao J.J., Wu F.Y., Liu Y.J., Li L., Lin Y.J., Kang Y.T., Peng Y.M., Liu Y.F., Wang C., Ma Z.S. (2024). Increase of PCSK9 Expression in Diabetes Promotes VEGFR2 Ubiquitination to Inhibit Endothelial Function and Skin Wound Healing. Sci. China Life Sci..

[85] Zhang M., Chen Y., Qiu Y., Sun J., He J., Liu Z., Shi J., Wei W., Wu G., Liang J. (2023). PCSK9 Promotes Hypoxia-Induced EC Pyroptosis by Regulating Smac Mitochondrion-Cytoplasm Translocation in Critical Limb Ischemia. JACC Basic Transl. Sci..

[86] Fang S., Yarmolinsky J., Gill D., Bull C.J., Perks C.M., Smith G.D., Gaunt T.R., Richardson T.G. (2023). Association between Genetically Proxied PCSK9 Inhibition and Prostate Cancer Risk: A Mendelian Randomisation Study. PLoS Med..

[87] WEISS T.S., BUECHLER C. (2025). Proprotein Convertase Subtilisin/Kexin Type 9 Induction in Hypercholesterinemic Patients with Primary and Metastatic Liver Tumors. Anticancer Res..

[88] ClinicalTrials.gov (2022). A Phase I Clinical Study to Evaluate the Safety, Tolerability, Pharmacokinetics and Preliminary Efficacy of Recombinant Humanized Anti-PCSK9 Monoclonal Antibody (JS002) Combined with Toripalimab in Patients with Advanced Cancer; Identifier: NCT05128539. NCT05128539.

[89] ClinicalTrials.gov (2024). A Phase II Study Bolstering Outcomes by Optimizing Immunotherapy Strategies with Evolocumab and Nivolumab in Patients with Metastatic Renal Cell Carcinoma (BOOST-RCC); Identifier: NCT06284564. NCT06284564.

[90] ClinicalTrials.gov (2023). A Phase II Study of PCSK9 Inhibitor Alirocumab and PD-1 Inhibitor Cemiplimab in Patients with Metastatic, Refractory To Prior Anti PD-1 Non-small Cell Lung Cancer: TOP2201; Identifier: NCT05553834. NCT05553834.

[91] Abdelwahed K.S., Siddique A.B., Mohyeldin M.M., Qusa M.H., Goda A.A., Singh S.S., Ayoub N.M., King J.A., Jois S.D., El Sayed K.A. (2020). Pseurotin A as a Novel Suppressor of Hormone Dependent Breast Cancer Progression and Recurrence by Inhibiting PCSK9 Secretion and Interaction with LDL Receptor. Pharmacol. Res..

[92] Xu W., Hu M., Lu X., Lao Y., Ma N., Wang Y., Li J., Chen X., Liu S., Liu J. (2024). Inhibition of PCSK9 Enhances the Anti-Hepatocellular Carcinoma Effects of TCR-T Cells and Anti-PD-1 Immunotherapy. Int. J. Biol. Sci..

[93] Lao M., Zhang X., Li Z., Sun K., Yang H., Wang S., He L., Chen Y., Zhang H., Shi J. (2025). Lipid Metabolism Reprograming by SREBP1-PCSK9 Targeting Sensitizes Pancreatic Cancer to Immunochemotherapy. Cancer Commun..

[94] Li Y., Zhou S., Fu W., Li X., Chen T., Le H., Xu Y., Tang Y., Mi P., Gao H. (2025). A Versatile Immunomodulated NanoCRISPR Converter Augments the Susceptibility and Visibility of Tumors to the Immune System. Proc. Natl. Acad. Sci. USA.

[95] Scalambra L., Ruzzi F., Pittino O.M., Semprini M.S., Cappello C., Angelicola S., Palladini A., Nanni P., Goksøyr L., Fougeroux C. (2025). Targeting PCSK9, through an Innovative CVLP-Based Vaccine, Enhanced the Therapeutic Activity of a CVLP-HER2 Vaccine in a Preclinical Model of HER2-Positive Mammary Carcinoma. J. Transl. Med..

[96] Ahmed N.A., Mohyeldin M.M., Ebrahim H.Y., McGehee O.C., Tarun M.T.I., El Sayed K.A. (2025). (−)-Oleuropein as a Novel Metastatic Castration-Resistant Prostate Cancer Progression and Recurrence Suppressor via Targeting PCSK9-LDLR Axis. Nutrients.

